# Inhibitors of α‐amylase and α‐glucosidase: Potential linkage for whole cereal foods on prevention of hyperglycemia

**DOI:** 10.1002/fsn3.1987

**Published:** 2020-11-04

**Authors:** Lingxiao Gong, Danning Feng, Tianxi Wang, Yuqing Ren, Yingli Liu, Jing Wang

**Affiliations:** ^1^ China‐Canada Joint Lab of Food Nutrition and Health (Beijing) Beijing Engineering and Technology Research Center of Food Additives Beijing Technology & Business University (BTBU) Beijing China

**Keywords:** enzyme inhibitor, hyperglycemia, peptides, polyphenols, polysaccharides

## Abstract

The strategy of reducing carbohydrate digestibility by controlling the activity of two hydrolyzing enzymes (α‐amylase and α‐glucosidase) to control postprandial hyperglycemia is considered as a viable prophylactic treatment of type 2 diabetes mellitus (T2DM). Thus, the consumption of foods rich in hydrolyzing enzyme inhibitors is recommended for diet therapy of diabetes. Whole cereal products have gained increasing interests for plasma glucose‐reducing effects. However, the mechanisms for whole cereal benefits in relation to T2DM are not yet fully understood, but most likely involve bioactive components. Cereal‐derived phenolic compounds, peptides, nonstarch polysaccharides, and lipids have been shown to inhibit α‐amylase and α‐glucosidase activities. These hydrolyzing enzyme inhibitors seem to make whole cereals become nutritional strategies in managing postmeal glucose for T2DM. This review presents an updated overview on the effects provided by cereal‐derived ingredients on carbohydrate digestibility. It suggests that there is some evidence for whole cereal intake to be beneficial in amelioration of T2DM through inhibiting α‐glucosidase and α‐amylase activities.

## Graphical abstract

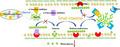

## 1 INTRODUCTION

Type 2 diabetes mellitus (T2DM) is commonly featured by postmeal or postprandial hyperglycemia. Nutritional strategies that designed to improve postprandial glycemia by reducing the glucose intake from the digestible carbohydrates were advised to the early‐stage diabetes patients before they were administered on pharmacologic treatment (Ch’ng et al., 2019). α‐Amylase (1,4‐α‐d‐glucan‐glucanohydrolase, EC 3.2. 1.1) and α‐glucosidase (EC 3.2.1.20) are the two key enzymes involved in the carbohydrate digestion process (Dona et al., 2010). Inhibitors of α‐amylase and α‐glucosidase, which slow the final stages of carbohydrate digestion and consequently preventing the entry of glucose into the circulation, are considered as a viable prophylactic treatment of hyperglycemia. However, synthetic and chemical α‐amylase and α‐glucosidase inhibitors have certain adverse effects such as causing gastrointestinal symptoms such as bloating, diarrhea, and abdominal pain (Chiasson et al., 2002). Natural glucosidase inhibitors from plants have become more important for the treatment of diabetes because of their less side effects and effectiveness.

Whole cereals are generally recommended for diabetic patients to control their blood glucose level. There have been many clinical and animal studies focused on the use of cereals and its components for the prevention of diabetes, especially based on glycemic Index values and hypoglycemic effects (Berglund et al., 1982; Brand‐Miller et al., 2003; Hallfrisch et al., 2003; Lundin et al., 2004). Although these beneficial effects are thought to be associated with dietary fiber intake, the actual underlying mechanism remains unclear. Besides dietary fiber, cereals are abundant in nutrients and bioactive ingredients for prevention and treatment of diabetes, such as polyphenol, anthocyanins, triterpenoids, saponins, polysaccharides, and peptides. It has been found that polysaccharides (Kim et al., 2015), phenols (Mcdougall & Stewart, 2005; Nyambe‐Silavwe et al., 2015; Tan & Chang, 2017), and proteins (Svensson et al., 2004) present in plants have an inhibitory effect on carbohydrate digestion enzymes. This paper presents a modern perspective on the inhibition of digestive enzymes by cereal constituent to encourage the design and development of whole cereal products for preventing T2DM.

## 2 α‐AMYLASE AND α‐GLUCOSIDASE INHIBITORS

Carbohydrate digestibility has been reported to relate to elevated postprandial blood glucose. One of the strategies to reduce postprandial hyperglycemia is to limit the activity of carbohydrate digestive enzymes in intestinal tract. α‐Amylase is the key enzyme that degrades the polymeric substrate into shorter oligomers by catalyzing the hydrolysis of α‐1,4‐glucan linkages present in starch, maltodextrins, and other related carbohydrates (Truscheit et al., 2010). α‐Glucosidase has been found on the brush border of human intestinal mucosal cells (including maltase, α‐dextrinase, and sucrase). This enzyme participates in the body's carbohydrate metabolism and cuts glucose from the nonreducing end of the polysaccharide by hydrolyzing the α‐1,4‐glycosidic bond. The dietary starch and other related carbohydrates are digested by α‐amylase to large number of maltose, which is further digested by α‐glucosidase to glucose to be absorbed in human intestine (Vocadlo & Davies, 2008). Therefore, strict control of postprandial blood glucose by inhibiting α‐glucosidase and α‐amylase is significant for the development of diabetes and the prevention and treatment of diabetic patients (Elbein, 1991; Tundis et al., 2010). α‐Amylase inhibitors (AIs) can act as carbohydrate blockers, limiting the digestibility and absorption of carbohydrate in the gastrointestinal diet (Horii et al., 1986). Clinically, AIs can be used to prevent diseases such as diabetes, hyperglycemia, hyperlipemia, and obesity. Moreover, in most cases, the inhibitory mechanism of protein to α‐amylase occurs by directly blocking the active centers of several subsites of the enzyme (Françoise, 2004). To determine the inhibition of α‐amylase, the most widely used method is the dinitrosalicylic acid (DNSA) assay, which is not selective for the reduction in oligosaccharide ends formed during hydrolysis (Bernfeld, 1955). The α‐glucosidase inhibitor (GI) inhibits α‐glucosidase activity by reversibly occupying α‐glucosidase and sugar‐binding sites, thereby reducing polysaccharide degradation, delaying intestinal absorption of carbohydrates, and achieving hypoglycemic effects. The most prominent feature of GIs is the inhibition of α‐glucosidase on the rate of intestinal carbohydrate decomposition (Larr, 2008; Seifarth et al., 1998), and it does not stimulate insulin secretion to lower blood sugar, thus not increasing the islet β‐cell burden. For α‐glucosidase, synthetic chromogenic molecular probes such as *p*‐nitrophenyl‐glucoside (pNPG) are widely used assays because of the ease of measurement. Natural GIs include iminosugars, thiosugars, flavonoids, alkaloids, and terpenes (Ghani, 2015).

## 3 WHOLE CEREAL PRODUCTS WITH LOWERING EFFECTS ON PLASMA GLUCOSE

The results of an umbrella review of meta‐analyses suggest that daily whole cereal intakes of 2 or 3 servings (30–45 g/day) can significantly reduce the incidence of developing T2DM and 1.5 servings of whole cereal per day significantly reduced both serum glucose and insulin concentrations (Mcrae, 2017). A meta‐analysis of randomized controlled trials including 17 studies and 212 subjects reported that the consumption of barley and barley products lowered postprandial glycemic response (Abumweis et al., 2016).

A series of experiments demonstrated that postprandial glucose response was improved when whole cereal products were consumed versus when refined cereals were consumed. For example, the consumption of breakfast meals with whole rye or whole wheat in healthy volunteers had lower early glucose responses (0–60 min) and incremental glucose peaks in comparison with white wheat bread. The whole rye and wheat products displayed a lower rate of starch hydrolysis (Rosén & Björck, 2011). Shukla and Srivastava (2014) reported that the glycemic index of refined wheat noodles incorporated with 30% finger millet was significantly lower (45.1) than refined wheat noodles (62.6) in ten normal female subjects aged 24–26 years. Several other studies also indicated the lowering effects of whole cereals and whole cereal products on plasma glucose (Berglund et al., 1982; Brand‐Miller et al., 2003; Lundin et al., 2004).

## 4 CEREAL ORIGINATED INHIBITORS AND INHIBITOR MECHANISMS

**FIGURE 1:**
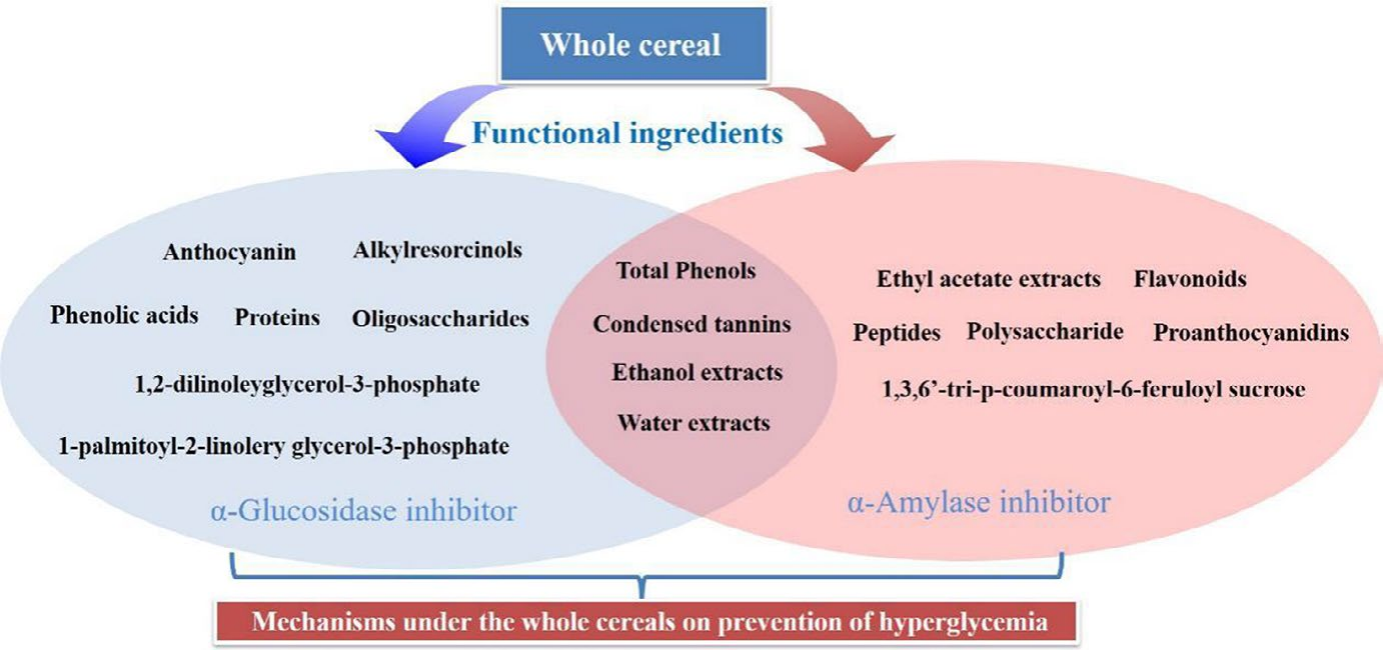
Cereal‐derived α‐amylase and α‐glucosidase inhibitors

Cereals are rich in polysaccharides, protein, and phenolic compounds, and are valuable resources for inhibitors of amylase and glucosidase (Figure 1). AIs have been found in cereals such as wheat, barley, sorghum, rye, and rice (Elbein, 1991; Mishra et al., 2017; Pradeep & Sreerama, 2015; Premakumara et al., 2013). The wheat AI isolated by Maeda et al. (1985) is the most studied inhibitors in cereals. It has been reported that taking a wheat amylase inhibitor for 9 weeks after meals can reduce postprandial amylase levels, delay carbohydrate digestion and absorption, and lower blood glucose levels without altering pancreas growth (Bernfeld, 1955). In this part, the recent advances made in discovery of starch hydrolase inhibitors from cereals are summarized.

### 4.1 Phenolic compounds

Starch hydrolase inhibitors from cereals have been reported for phenolic acids, tannins, anthocyanins, and flavonoids (Table 1). Most of the reported studies used crude phenolic extracts, including soluble and bound forms. The phenolic compounds from corn, rice, barley, sorghum, millet, and quinoa (Pradeep & Sreerama, 2015; Rosén & Björck, 2011; Shukla & Srivastava, 2014) were reported to be potent inhibitors of α‐amylase and α‐glucosidase. The soluble and bound phenolic extracts of whole cereals and their milled fraction inhibited the activities of α‐amylase and α‐glucosidase in a dose‐dependent manner (Qin et al., 2013). The enzyme inhibitory activities of cereal phenolic extracts depend on the cereal types and processing methods. For example, the phenolic extracts of little millet cultivars had superior inhibition of both α‐amylase and α‐glucosidase than those of foxtail millet cultivars. Among the evaluated cultivars, the soluble and bound fractions of foxtail millet cultivar CO7 (IC_50_, 22.37 and 57.26 μg/ml) and the little millet cultivar CO4 of (IC_50_, 18.97 and 55.69 μg/ml) showed strong inhibition of α‐glucosidase (Pradeep et al., 2018). HPLC analysis of phenolic extracts revealed variations in individual phenolic acid composition among the evaluated samples. Naringenin, kaempferol, luteolin glycoside, apigenin, (+)‐catechin/(−)‐epicatechin, daidzein, caffeic acid, ferulic acid, and syringic acid from cereals are considered as the enzyme inhibitors (Shobana et al., 2009). Caffeic, ferulic, and sinapic acids were found as the predominant phenolic acids in soluble fractions, while ferulic and *p*‐coumaric acids were abundant in bound fractions. Quercetin was the most abundant flavonoid presented in all the fractions. Quercetin, and ferulic and p‐coumaric acids are reported to have high α‐glucosidase inhibitory activity by mixed noncompetitive inhibition (Adisakwattana et al., 2009; Li et al., 2009). The results of Mishra et al. (2017) had shown that organic rye varieties have higher ferulic acid content and α‐amylase inhibitory activity, while traditional rye varieties have higher catechin content and α‐glucosidase inhibitory activity.

**TABLE 1:** A summary of newly discovered phenolic compounds as α‐glucosidase and α‐amylase inhibitors

No.	Variety	Part used	Active compounds	Inhibited enzyme	Enzyme origin	Method	IC_50_	Reference
1	Buckwheat	Bran	Flavonoids	α‐Glucosidase	Saccharomyces cerevisiae	pNPG		Li et al. (2009)
2	Millet	Seed coat	Phenolic extracts	α‐Amylase	Porcine pancreatic	DNSA	16.9 μg/ml	Shobana et al. (2009)
				α‐Glucosidase	Rats’ intestine	pNPG	23.5 μg/ml	
3	Black rice	Kernel	Anthocyanin	α‐Glucosidase	Rat intestine	p‐Nitrophenyl‐α‐d‐glucopyranoside	13.56 ± 1.2 mg/ml	Yao et al. (2010)
	Purple rice	Kernel	Anthocyanin	α‐Glucosidase	Rat intestine	p‐Nitrophenyl‐α‐d‐glucopyranoside	475.14 ± 25.46 mg/ml	
	Purple corn	Kernel	Anthocyanin	α‐Glucosidase	Rat intestine	p‐Nitrophenyl‐α‐d‐glucopyranoside	833.33 ± 56.31 mg/ml	
	Black barley	Kernel	Anthocyanin	α‐Glucosidase	Rat intestine	p‐Nitrophenyl‐α‐d‐glucopyranoside	>1,000 mg/ml	
	Red rice	Kernel	Anthocyanin	α‐Glucosidase	Rat intestine	p‐Nitrophenyl‐α‐d‐glucopyranoside	>1,000 mg/ml	
4	Proanthocyanidin‐rich sumac sorghum	Bran	Simple flavonoids and proanthocyanidins	α‐Glucosidase	Porcine pancreas	EnzChek Ultra Amylase Assay Kit	1.4 μg/ml	Hargrove et al. (2011)
	Proanthocyanidin‐free black sorghum	Bran	Simple flavonoids and proanthocyanidins	α‐Glucosidase	Porcine pancreas	EnzChek Ultra Amylase Assay Kit	11.4 μg/ml	
	Sumac sorghum	Bran	Simple flavonoids and proanthocyanidins	α‐Glucosidase	Porcine pancreas	EnzChek Ultra Amylase Assay Kit	12.1 μg/ml	
	Black sorghum	Bran	Simple flavonoids and proanthocyanidins	α‐Glucosidase	Porcine pancreas	EnzChek Ultra Amylase Assay Kit	18.8 μg/ml	
5	Buckwheat	Seed	Total phenols	α‐Glucosidase	Rat intestine	p‐Nitrophenyl‐α‐d‐glucopyranoside		Qin et al. (2013)
6	Rice of Sri Lankan	Bran	Ethanol extracts	α‐Amylase	Bacillus amyloliquefaciens	DNSA		Premakumara et al. (2013)
				α‐Glucosidase	Rice	pNPG		
7	Sorghum	Bran	Condensed tannins	α‐Amylase	Porcine	DNSA	554.5 μg/ml (IC_50_ of acarbose = 3.1 μg/ml)	Links et al. (2015)
				α‐Glucosidase	Yeast	pNPG	0.4 μg/ml (IC_50_ of acarbose = 8,464.0 μg/ml)	
8	Barnyard millet	Raw grains	Phenolic extracts	α‐Amylase	Porcine pancreatic	DNSA	32.59 ± 1.04 μg/ml	Pradeep and Sreerama (2015)
				α‐Glucosidase	Rats’ intestine	pNPG	18.60 ± 0.83 μg/ml	
		Germinated grains	Phenolic extracts	α‐Amylase	Porcine pancreatic	DNSA	17.26 μg/ml	
				α‐Glucosidase	Rats’ intestine	pNPG	7.46 μg/ml	
		Steamed grains	Phenolic extracts	α‐Amylase	Porcine pancreatic	DNSA	59.21 μg/ml	
				α‐Glucosidase	Rats’ intestine	pNPG	42.71 μg/ml	
		Microwave grains	Phenolic extracts	α‐Amylase	Porcine pancreatic	DNSA	49.7 μg/ml	
				α‐Glucosidase	Rats’ intestine	pNPG	36.81 μg/ml	
	Foxtail millet	Raw grains	Phenolic extracts	α‐Amylase	Porcine pancreatic	DNSA	67.38 ± 3.5 μg/ml	
				α‐Glucosidase	Rats’ intestine	pNPG	19.21 ± 1.42 μg/ml	
		Germinated grains	Phenolic extracts	α‐Amylase	Porcine pancreatic	DNSA	41.81 μg/ml	
				α‐Glucosidase	Rats’ intestine	pNPG	8.61 μg/ml	
		Steamed grains	Phenolic extracts	α‐Amylase	Porcine pancreatic	DNSA	108.64 μg/ml	
				α‐Glucosidase	Rats’ intestine	pNPG	51.26 μg/ml	
		Microwave grains	Phenolic extracts	α‐Amylase	Porcine pancreatic	DNSA	98.62 μg/ml	
				α‐Glucosidase	Rats’ intestine	pNPG	61.43 μg/ml	
	Proso millet	Raw grains	Phenolic extracts	α‐Amylase	Porcine pancreatic	DNSA	34.15 ± 1.87 μg/ml	
				α‐Glucosidase	Rats’ intestine	pNPG	29.47 ± 1.47 μg/ml	
		Germinated grains	Phenolic extracts	α‐Amylase	Porcine pancreatic	DNSA	20.04 μg/ml	
				α‐Glucosidase	Rats’ intestine	pNPG	16.09 μg/ml	
		Steamed grains	Phenolic extracts	α‐Amylase	Porcine pancreatic	DNSA	94.37 μg/ml	
				α‐Glucosidase	Rats’ intestine	pNPG	66.19 μg/ml	
		Microwave grains	Phenolic extracts	α‐Amylase	Porcine pancreatic	DNSA	84.14 μg/ml	
				α‐Glucosidase	Rats’ intestine	pNPG	84.62 μg/ml	
9	Quinoa	Whole grains	Phenolic extracts	α‐Amylase	Porcine pancreatic	DNSA	163.52 ± 2.5 μg/ml (IC50 of acarbose = 7.21 ± 0.4 μg/ml)	Hemalatha et al. (2016)
				α‐Glucosidase	Rats’ intestine	pNPG	72.36 ± 1.5 μg/ml ((IC50 of acarbose = 83.65 ± 4.7 μg/ml)	
		Hulls	Phenolic extracts	α‐Amylase	Porcine pancreatic	DNSA	148.23 ± 4.6 μg/ml (IC50 of acarbose = 7.21 ± 0.4 μg/ml)	
				α‐Glucosidase	Rats’ intestine	pNPG	68.14 ± 3.8 μg/ml ((IC50 of acarbose = 83.65 ± 4.7 μg/ml)	
		Dehulled grain	Phenolic extracts	α‐Amylase	Porcine pancreatic	DNSA	179.5 ± 3.8 μg/ml (IC50 of acarbose = 7.21 ± 0.4 μg/ml)	
				α‐Glucosidase	Rats’ intestine	pNPG	116.2 ± 5.3 μg/ml ((IC50 of acarbose = 83.65 ± 4.7 μg/ml)	
		Milled grain	Phenolic extracts	α‐Amylase	Porcine pancreatic	DNSA	241.36 ± 9.8 μg/ml (IC50 of acarbose = 7.21 ± 0.4 μg/ml)	
				α‐Glucosidase	Rats’ intestine	pNPG	182.01 ± 2.0 μg/ml ((IC50 of acarbose = 83.65 ± 4.7 μg/ml)	
		Bran	Phenolic extracts	α‐Amylase	Porcine pancreatic	DNSA	108.68 ± 3.1 μg/ml (IC50 of acarbose = 7.21 ± 0.4 μg/ml)	
				α‐Glucosidase	Rats’ intestine	pNPG	62.1 ± 3.9 μg/ml ((IC50 of acarbose = 83.65 ± 4.7 μg/ml)	
10	7 varieties of cereals	Whole grain	Methanol extracts	α‐Amylase	Porcine pancreatic	DNSA		Donkor et al. (2017)
				α‐Glucosidase		pNPG		
11	Yellow corn	Whole grain	Bound polyphenol	α‐Amylase	Porcine pancreatic	DNSA		Gong et al. (2018)
			Free polyphenol	α‐Glucosidase		pNPG		
12	Rye	Whole grain	Phenolic extracts	α‐Amylase	Porcine pancreatic	DNSA		Mishra et al. (2017)
				α‐Glucosidase	Baker's yeast	pNPG		
13	Foxtail millet	Whole grains	Soluble phenolic extracts	α‐Amylase	Porcine pancreatic	DNSA	67.28 ± 0.69 μg/ml	Pradeep and Sreerama (2017)
				α‐Glucosidase	Rats’ intestine	pNPG	19.87 ± 0.69 μg/ml	
			Bound phenolic extracts	α‐Amylase	Porcine pancreatic	DNSA	98.28 ± 1.69 μg/ml	
				α‐Glucosidase	Rats’ intestine	pNPG	52.64 ± 0.85 μg/ml	
		Dehulled grains	Soluble phenolic extracts	α‐Amylase	Porcine pancreatic	DNSA	81.85 ± 0.43 μg/ml	
				α‐Glucosidase	Rats’ intestine	pNPG	37.68 ± 0.31 μg/ml	
			Bound phenolic extracts	α‐Amylase	Porcine pancreatic	DNSA	115.63 ± 1.02 μg/ml	
				α‐Glucosidase	Rats’ intestine	pNPG	110.25 ± 0.91 μg/ml	
		Pearled grains	Soluble phenolic extracts	α‐Amylase	Porcine pancreatic	DNSA	109.91 ± 1.82 μg/ml	
				α‐Glucosidase	Rats’ intestine	pNPG	67.60 ± 0.41 μg/ml	
			Bound phenolic extracts	α‐Amylase	Porcine pancreatic	DNSA	162.56 ± 1.53 μg/ml	
				α‐Glucosidase	Rats’ intestine	pNPG	198.64 ± 1.17 μg/ml	
		Hull	Soluble phenolic extracts	α‐Amylase	Porcine pancreatic	DNSA	32.29 ± 0.59 μg/ml	
				α‐Glucosidase	Rats’ intestine	pNPG	10.28 ± 0.63 μg/ml	
			Bound phenolic extracts	α‐Amylase	Porcine pancreatic	DNSA	41.74 ± 0.64 μg/ml	
				α‐Glucosidase	Rats’ intestine	pNPG	35.21 ± 0.42 μg/ml	
		Bran	Soluble phenolic extracts	α‐Amylase	Porcine pancreatic	DNSA	39.64 ± 0.57 μg/ml	
				α‐Glucosidase	Rats’ intestine	pNPG	12.41 ± 0.39 μg/ml	
			Bound phenolic extracts	α‐Amylase	Porcine pancreatic	DNSA	54.29 ± 0.81 μg/ml	
				α‐Glucosidase	Rats’ intestine	pNPG	35.26 ± 0.37 μg/ml	
	Little millet	Whole grains	Soluble phenolic extracts	α‐Amylase	Porcine pancreatic	DNSA	61.91 ± 1.07 μg/ml	
				α‐Glucosidase	Rats’ intestine	pNPG	17.22 ± 0.48 μg/ml	
			Bound phenolic extracts	α‐Amylase	Porcine pancreatic	DNSA	84.31 ± 1.07 μg/ml	
				α‐Glucosidase	Rats’ intestine	pNPG	49.22 ± 0.72 μg/ml	
		Dehulled grains	Soluble phenolic extracts	α‐Amylase	Porcine pancreatic	DNSA	74.97 ± 0.76 μg/ml	
				α‐Glucosidase	Rats’ intestine	pNPG	25.53 ± 0.17 μg/ml	
			Bound phenolic extracts	α‐Amylase	Porcine pancreatic	DNSA	107.45 ± 1.32 μg/ml	
				α‐Glucosidase	Rats’ intestine	pNPG	89.26 ± 1.65 μg/ml	
		Pearled grains	Soluble phenolic extracts	α‐Amylase	Porcine pancreatic	DNSA	89.46 ± 0.71 μg/ml	
				α‐Glucosidase	Rats’ intestine	pNPG	38.72 ± 0.21 μg/ml	
			Bound phenolic extracts	α‐Amylase	Porcine pancreatic	DNSA	131.71 ± 1.30 μg/ml	
				α‐Glucosidase	Rats’ intestine	pNPG	115.71 ± 1.44 μg/ml	
		Hull	Soluble phenolic extracts	α‐Amylase	Porcine pancreatic	DNSA	27.21 ± 0.85 μg/ml	
				α‐Glucosidase	Rats’ intestine	pNPG	9.27 ± 0.12 μg/ml	
			Bound phenolic extracts	α‐Amylase	Porcine pancreatic	DNSA	38.27 ± 0.49 μg/ml	
				α‐Glucosidase	Rats’ intestine	pNPG	31.34 ± 0.37 μg/ml	
		Bran	Soluble phenolic extracts	α‐Amylase	Porcine pancreatic	DNSA	33.34 ± 0.58 μg/ml	
				α‐Glucosidase	Rats’ intestine	pNPG	12.32 ± 0.16 μg/ml	
			Bound phenolic extracts	α‐Amylase	Porcine pancreatic	DNSA	46.94 ± 0.77 μg/ml	
				α‐Glucosidase	Rats’ intestine	pNPG	33.83 ± 0.83 μg/ml	
14	Foxtail millet (CO5)	Whole grains	Soluble phenolic extracts	α‐Amylase	Porcine pancreatic	DNSA	79.61 ± 2.58 μg/ml (IC_50_ of acarbose = 10.54 ± 1.06 mg/ml)	Pradeep and Sreerama (2018)
				α‐Glucosidase	Rats’ intestine	pNPG	23.54 ± 0.53 μg/ml (IC_50_ of acarbose = 91.38 ± 6.20 mg/ml)	
			Bound phenolic extracts	α‐Amylase	Porcine pancreatic	DNSA	112.62 ± 3.46 μg/ml (IC_50_ of acarbose = 10.54 ± 1.06 mg/ml)	
				α‐Glucosidase	Rats’ intestine	pNPG	60.17 ± 1.50 μg/ml (IC_50_ of acarbose = 91.38 ± 6.20 mg/ml)	
	Foxtail millet( CO6)	Whole grains	Soluble phenolic extracts	α‐Amylase	Porcine pancreatic	DNSA	74.66 ± 2.37 μg/ml (IC_50_ of acarbose = 10.54 ± 1.06 mg/ml)	
				α‐Glucosidase	Rats’ intestine	pNPG	26.81 ± 0.45 μg/ml (IC_50_ of acarbose = 91.38 ± 6.20 mg/ml)	
			Bound phenolic extracts	α‐Amylase	Porcine pancreatic	DNSA	117.33 ± 3.67 μg/ml (IC_50_ of acarbose = 10.54 ± 1.06 mg/ml)	
				α‐Glucosidase	Rats’ intestine	pNPG	66.29 ± 2.47 μg/ml (IC_50_ of acarbose = 91.38 ± 6.20 mg/ml)	
	Foxtail millet (CO7)	Whole grains	Soluble phenolic extracts	α‐Amylase	Porcine pancreatic	DNSA	69.92 ± 1.25 μg/ml (IC_50_ of acarbose = 10.54 ± 1.06 mg/ml)	
				α‐Glucosidase	Rats’ intestine	pNPG	22.37 ± 0.64 μg/ml (IC_50_ of acarbose = 91.38 ± 6.20 mg/ml)	
			Bound phenolic extracts	α‐Amylase	Porcine pancreatic	DNSA	101.55 ± 2.85 μg/ml (IC_50_ of acarbose = 10.54 ± 1.06 mg/ml)	
				α‐Glucosidase	Rats’ intestine	pNPG	57.26 ± 1.26 μg/ml (IC_50_ of acarbose = 91.38 ± 6.20 mg/ml)	
	Little millet (CO2)	Whole grains	Soluble phenolic extracts	α‐Amylase	Porcine pancreatic	DNSA	67.26 ± 1.79 μg/ml (IC_50_ of acarbose = 10.54 ± 1.06 mg/ml)	
				α‐Glucosidase	Rats’ intestine	pNPG	20.17 ± 0.61 μg/ml (IC_50_ of acarbose = 91.38 ± 6.20 mg/ml)	
			Bound phenolic extracts	α‐Amylase	Porcine pancreatic	DNSA	96.22 ± 3.42 μg/ml (IC_50_ of acarbose = 10.54 ± 1.06 mg/ml)	
				α‐Glucosidase	Rats’ intestine	pNPG	58.65 ± 1.61 μg/ml (IC_50_ of acarbose = 91.38 ± 6.20 mg/ml)	
	Little millet (CO3)	Whole grains	Soluble phenolic extracts	α‐Amylase	Porcine pancreatic	DNSA	69.87 ± 2.05 μg/ml (IC_50_ of acarbose = 10.54 ± 1.06 mg/ml)	
				α‐Glucosidase	Rats’ intestine	pNPG	21.85 ± 0.75 μg/ml (IC_50_ of acarbose = 91.38 ± 6.20 mg/ml)	
			Bound phenolic extracts	α‐Amylase	Porcine pancreatic	DNSA	98.47 ± 2.64 μg/ml (IC_50_ of acarbose = 10.54 ± 1.06 mg/ml)	
				α‐Glucosidase	Rats’ intestine	pNPG	56.11 ± 2.03 μg/ml (IC_50_ of acarbose = 91.38 ± 6.20 mg/ml)	
	Little millet (CO4)	Whole grains	Soluble phenolic extracts	α‐Amylase	Porcine pancreatic	DNSA	64.32 ± 1.95 μg/ml (IC_50_ of acarbose = 10.54 ± 1.06 mg/ml)	
				α‐Glucosidase	Rats’ intestine	pNPG	18.97 ± 0.43 μg/ml (IC_50_ of acarbose = 91.38 ± 6.20 mg/ml)	
			Bound phenolic extracts	α‐Amylase	Porcine pancreatic	DNSA	93.89 ± 1.75 μg/ml (IC_50_ of acarbose = 10.54 ± 1.06 mg/ml)	
				α‐Glucosidase	Rats’ intestine	pNPG	55.69 ± 1.59 μg/ml (IC_50_ of acarbose = 91.38 ± 6.20 mg/ml)	

The enzyme inhibition potency of individual phenolic compounds through mixed, uncompetitive, and competitive type is highly correlated with their structures (Di Stefano et al., 2018; Kim et al., 2019; Malunga et al., 2018; Tadera et al., 2006). Hydroxycinnamic acids are reported to be more potent on inhibition of α‐glucosidase compared with their corresponding hydroxybenzoic acid derivatives. The structure–activity relationship suggests that the number of hydroxyl and methoxy groups present in the aromatic ring phenolic acids decides the inhibitory activity (Malunga et al., 2018). Furthermore, flavonoids appeared to have a better α‐glucosidase inhibitory activity than phenolic acids due to the additional hydroxyl groups in flavone skeleton, which is most likely responsible for the more pronounced inhibitory activity (Di Stefano et al., 2018; Tadera et al., 2006). Additionally, proper glucoside substitutions may boost the enzyme inhibition activities and types due to increased number of total aromatic hydroxyl groups (Şöhretoğlu et al., 2018). For example, the hydroxy (–OH) groups at C‐3 position of ring C, C‐3’ and C‐4’ position of ring B, and the glucoside substitutions at the C‐3 position of ring C were crucial for the enzyme inhibition activities of flavonols. Molecular docking studies revealed that phenolic compounds bind at both the active sites and allosteric sites, resulting in structural changes and activity inhibitors (Kim et al., 2019; Martinez‐Gonzalez et al., 2019). Hydrogen bonds, hydrophobic interactions, and van der Waals interactions are the predominant force involved in the complexation of the phenolic compounds with enzymes (Di Stefano et al., 2018; Martinez‐Gonzalez et al., 2019).

Polyphenols with higher polymerization also have inhibition effects on the enzymes. Pigmented cereals always gained attentions due to their health benefits associated with anthocyanins (Pei‐Ni et al., 2006). Premakumara et al. (2013) screened the most resistant varieties of α‐amylase in 70% ethanol extracts from 35 varieties of rice (red and white) in Sri Lankan. The results showed that the anti‐amylase activity of the red wheat bran extract was significantly higher than the white wheat bran extract. The traditional red rice varieties Masuran, Sudu Heenati, etc., all showed significant anti‐amylase activity in a dose‐dependent manner. Yao et al. (2010) studied Chinese colored cereals, including red, purple, black rice, purple corn, black barley, and black soybeans. Among the Chinese colored cereals studied, black rice (IC_50_ = 13.56 ± 1.2 mg/ml) has the highest total anthocyanin content, total phenolic content, and α‐glucosidase inhibitory activity. Anthocyanin in purple rice (IC_50_ = 475.14 ± 25.46 mg/ml) has stronger inhibitory activity against α‐glucosidase than proanthocyanidins in red rice (IC_50_ > 1,000 mg/ml). On the other hand, Hargrove et al. (2011) compared the inhibition of α‐amylase by monoflavonoids and proanthocyanidins in Sorghum bicolor bran extract. The results showed that the extract of sumac sorghum bran rich in proanthocyanidins (IC_50_ = 1.4 μg/ml) had a stronger inhibitory effect on α‐amylase than the extract of sorghum bran (IC_50_ = 11.4 μg/ml) without procyanidins. In addition, flavonoids have higher IC_50_ values than proanthocyanidins. Links et al. (2015) prepared a highly efficient sorghum‐condensed tannins (SCT) from sorghum. The results showed that SCT was a better α‐glucosidase inhibitor (IC_50_ = 0.4 μg/ml) compared with acarbose (IC_50_ = 8,464.0 μg/ml). SCT also had a certain inhibitory effect on α‐amylase. The effect of kafirin microparticles (KEMS) as an oral administration system for SCT has potential hypoglycemic effect. Lignin can also be used as novel α‐amylase inhibitor. The molecular docking studies indicated that the major binding sites are –OH in G units and β‐O‐4 structure of lignin on α‐amylase molecule (Fan et al., 2019).

Different processing methods significantly affected the total phenolics, individual phenolic compounds, and enzyme inhibitory properties of cereals (Donkor et al., 2012; Pradeep & Sreerama, 2015). Germinated millets with higher phenolic compound levels showed highest inhibitory activities toward both the enzymes that their untreated, steamed, and microwaved treated cereal counterparts (Pradeep & Sreerama, 2015). Similar results were also obtained in germinated wheat, brown rice, barley, sorghum, oat, rye, and buckwheat cereals (Donkor et al., 2012). In the recent study of Gong et al. (2018), germination combined with extrusion on free and bound phenolic compound extracts of whole cereal corn increased the anti‐α‐glucosidase activity by 221 and 40%, and increased the anti‐α‐amylase activity by 105 and 108%. In the study of Qin et al. (2013), soaked tartary buckwheat had increased quercetin, kaempferol, total flavonoid, and total phenolic compound contents which were responsible for the highest α‐glucosidase inhibitory activity as compared with raw, steamed, and dried tartary buckwheat. The findings of Irondi et al. (2019) demonstrated that both the α‐glucosidase and α‐amylase inhibitory activities of sorghum decreased due to roasting, in contradictory with the report of Kunyanga et al. (2011) that indicated an increase in enzyme inhibitory activities of pearl millet. The parallel changes in phenolic compound levels and enzyme inhibitory activity with different processing methods suggest that phenolic compounds might be the major enzyme inhibitors in the cereals. Additionally, phenolic compounds are mainly concentrated in the pericarp, hull bran, and aleurone layers of whole cereals which may promote their contribution to the enzyme inhibitory activities. The phenolic extracts of these fractions usually displayed strong inhibition toward α‐glucosidase and α‐amylase compared with other fractions of whole cereals (Hemalatha et al., 2016; Pradeep & Sreerama, 2017). Moreover, the digestibility of phenolic compounds plays a key role in their enzyme inhibitory activity in the small intestine. Unencapsulated sorghum‐condensed tannins had minimal α‐amylase inhibition and no α‐glucosidase inhibition after pepsin and trypsin–chymotrypsin digestion (Links et al., 2015). Hence, technologies that ensure bioaccessibility of phenolic compounds in the target site (small intestine) are needed in improving anti‐enzyme activities of whole cereals.

### 4.2 Peptides

Some of the bioactive peptides generated from cereal proteins by enzymatic, or chemical hydrolysis and fermentation, have been reported to exhibit enzyme‐inhibiting activities (Table 2). The inhibition activity of peptides from rice bran protein on α‐amylase, ranged from 6.9 to 56.1 μg acarbose equivalent mg^‐1^ protein, was generally correlated with the degree of protein hydrolysis (Uraipong & Zhao, 2015). Moreover, different fractions (albumin, globulin, prolamin, and glutelin) of rice bran proteins, which were subjected to different protease hydrolysis (Alcalase, Neutrase, Flavourzyme, and Protamax), resulted in different activities. In general, highest inhibition activities were found with albumin and glutelin hydrolysates produced by Protamax‐ and Alcalase‐catalyzed hydrolysis. The results of this in vitro study highlight that the bioactivity of peptides in the hydrolysates is dependent on the proteolytic enzyme used. Accordingly, hydrolysates generated by 14 different enzymes from barley and brewers’ spent grain protein were evaluated for the α‐glucosidase and α‐amylase inhibition activities by Connolly et al. (2014). The tryptic hydrolysate resulted in the highest inhibition of α‐glucosidase, which had increased from 12.43% inhibition for unhydrolyzed protein‐enriched isolates to 66.81% at 7.5 mg/ml. For α‐amylase inhibition, the unhydrolyzed isolates inhibited by 8.08% to 13.35% with the concentration increased from 2.5 to 7.5 mg/ml. However, no significant increases were found for all 14 hydrolysates. Additionally, the α‐amylase and α‐glucosidase inhibitory peptides may also be produced during digestion. Peptides released from quinoa during the in vitro duodenal phase showed the highest inhibitory effects, which reaching an IC_50_ value of 0.19 mg protein/ml for α‐amylase inhibition and 1.75 mg protein/ml for α‐glucosidase inhibition, respectively (Vilcacundo et al., 2017).

**TABLE 2:** A summary of newly discovered peptides as α‐glucosidase and α‐amylase inhibitors

No.	Variety	Part used	Active compounds	Inhibited enzyme	Enzyme origin	Method	IC50	Reference
1	Rice	Bran	Protein and peptide	α‐Amylase	Bacillus amyloliquefaciens	DNSA		Uraipong and Zhao (2015)
				α‐Glucosidase	Saccharomyces cerevisiae	pNPG		
2	Pale brewers' spent grain		Protein	α‐Amylase	Porcine pancreatic	DNSA		Connolly et al. (2014)
				α‐Glucosidase	Rats’ intestine	pNPG		
3	Quinoa	Seed	Protein	α‐Amylase	Porcine pancreatic	DNSA		Vilcacundo et al. (2017)
				α‐Glucosidase	Rats’ intestine	Glucose/Glucose Oxidase Assay Kit		

The structure–activity relationships were studied for several identified peptides. The most potent α‐glucosidase inhibitory peptide identified is LQAFEPLR (IC_50_ = 35.67 μg/ml) derived from oat globulin by trypsin hydrolysis. As demonstrated by Di Stefano et al. (2018), α‐glucosidase inhibitory activity appears to occur more for peptides containing serine, threonine, tyrosine, lysine, or arginine at the N‐terminal, and a proline residue closer to the C‐terminal with methionine or alanine occupying the C‐terminal position. The roles of hydrophobic amino acids on the inhibition of α‐glucosidase were confirmed by Vilcacundo et al. (2017). Potential α‐glucosidase inhibitory peptides identified in quinoa after digestion were IQAEGGLT and DKKYPK. Peptide IQAEGGLT containing three hydrophobic residues showed potent inhibitory activity toward α‐glucosidase via hydrophobic interaction. In case of α‐amylase inhibitory peptides, the aromatic–aromatic interactions between the enzyme residues and peptide arising from hydrogen bonds, and electrostatic and Van der Waals interactions, which may form a sliding barrier via a hydrogen bonding with the residues of the active/substrate‐binding region, are critically for the inhibitory activity (Siow & Gan, 2016, 2017). The α‐amylase has numbers of aromatic residues including phenylalanine, tryptophan, and tyrosine.

### 4.3 Nonstarch polysaccharides

Nonstarch polysaccharides with α‐glucosidase and α‐amylase inhibitory activities have been identified in barley, wheat, buckwheat, and corn silk (Table 3). The barley polysaccharide exhibited a noncompetitively inhibitory process toward α‐glucosidase with IC_50_ at 22.49 mg/ml. The sulfation can significantly raise the enzyme inhibitory activity with increases in dose and the degree of substitution of sulfate group. However, the mechanism of sulfated polysaccharides against α‐glucosidase was reversible as a mixed one (Qian et al., 2015). The oligosaccharide from barley malt defined as α‐pyran glucosan composed of four glucoses with (1 → 3) linkage was an effective α‐glucosidase inhibitors as acarbose (Shelat et al., 2011). The IC_50_ of crude oligosaccharides, pure oligosaccharides, and acarbose was 1.30, 0.48, and 0.26 mg/ml, respectively. A novel purified neutral polysaccharide (TBP‐II, 26 kDa) from buckwheat was reported to exhibit α‐glucosidase inhibitory activity (Wang et al., 2016). TBP‐II was mainly consisted of galactose, arabinose, xylose, and glucose with a molar ratio of 0.7:1:6.3:74.2. The backbone of TBP‐II was composed of (1 → 4)‐linked‐d‐glucopyranosyl (*Glcp)*, while the branches comprised of (1 → 3)‐linked‐d‐glucopyranosyl (*Glcp*), (1 → 6)‐linked‐d‐galactopyranosyl (*Galp*), and (1 → 2,4)‐linked‐d‐rhamnopyranosyl (*Rhap*). TBP‐II exhibited an excellent inhibitory activity on α‐glucosidase, which was superior to acarbose and crude polysaccharide, and the percentage inhibition depended on the concentration of polysaccharides.

**TABLE 3:** A summary of newly discovered nonstarch polysaccharides as α‐glucosidase and α‐amylase inhibitors

No.	Variety	Part used	Active compounds	Inhibited enzyme	Enzyme origin	Method	IC50	Reference
1	Corn	Silk	Carboxymethylated polysaccharide	α‐Amylase		DNSA	5.33 mg/ml (IC50 of acarbose = 91.38 ± 6.20 mg/ml)	Shuhan et al. (2013)
			Sulfated polysaccharide				8.54 mg/ml	
			Raw polysaccharide				10.07 mg/ml	
			Acetylated polysaccharide				10.31 mg/ml	
2	Barely	Seed	Oligosaccharides(BP)	α‐Glucosidase		pNPG	22.49 mg/ml	Qian et al. (2015)
3	Fagopyrum tartaricum	Seed	Neutral polysaccharide	α‐Glucosidase		Glucose oxidase method		Wang et al. (2016)

In case of α‐amylase, cellulose (either purified or as a component of wheat bran) was demonstrated to bind α‐amylase and inhibit the activity of the enzyme through a mixed‐type inhibition mechanism (Sushil et al., 2015). Cereal arabinoxylan and β‐glucan can impair diffusion of the polymer probes similar in size to α‐amylase, which slowed starch hydrolysis in the small intestine (Shelat et al., 2010, 2011). The α‐amylase inhibitory activities among water‐soluble corn silk polysaccharides and their sulfated, acetylated, and carboxymethylated derivatives were compared by Shuhan et al. (2013). The carboxymethylated polysaccharide, which exhibited the highest inhibitory activity among the four polysaccharides samples, had a high solubility, a narrow molecular weight distribution, and a hyperbranched conformation. The IC_50_ of acarbose, carboxymethylated, sulfated, raw, and acetylated derivatives was 2.51, 5.33, 8.54, 10.07, and 10.31 mg/ml. However, limited work has been conducted on the structure–function relationship of nonstarch polysaccharides against α‐glucosidase or α‐amylase.

### 4.4 Lipids

**FIGURE 2:**
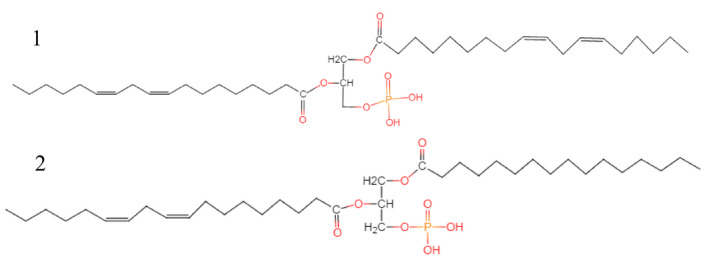
Structure of 1,2‐dilinoleylglycerol‐3‐phosphate (1) and 1‐palmitoyl‐2‐linoleoyl glycerol‐3‐phosphate (2)

Lipids derived from cereals are another potential α‐glucosidase and α‐amylase inhibitors (Table 4). The Soxhlet hexane and ethyl acetate extracts of wheat bran were effective inhibitors of α‐glucosidase in vitro (Liu, 2009). The isolated phosphatidic acids in wheat germ, 1,2‐dilinoleylglycerol‐3‐phosphate and 1‐palmitoyl‐2‐linoleoyl glycerol‐3‐phosphate (Figure 2), showed the highest α‐glucosidase inhibitory activity among the test lipids with the IC_50_ of 38.9 and 47.9 μM (Liu et al., 2011). The structure–activity relationship studies suggested that the unsaturated fatty acids and phosphate group in the glycerides were significant structural requirements for the inhibitory activity. Alkylresorcinols are another important components that are responsible for the α‐glucosidase inhibitory activity of wheat bran lipids (Tu et al., 2013). Alkylresorcinols showed IC_50_ of 37.58 μg/ml by noncompetitive type of inhibition. It has been reported that the important biological role of alkylresorcinols is to directly regulate enzyme activity, such as inhibition of acetylcholinesterase, and the inhibitory activity is affected by the length of the alkyl side chain (Athukorala et al., 2010; Stasiuk et al., 2008). However, its effects on inhibition of α‐glucosidase still require further investigation. Additionally, there is some evidence that fatty acids, saponins, and terpenes found in fruits, vegetables, and mushrooms contribute to the in vitro α‐glucosidase and α‐amylase inhibition activities of hexane extracts (Papoutsis et al., 2020). However, limited work has been conducted on elucidation of the α‐glucosidase and α‐amylase inhibition activities of different lipophilic compounds found in cereals. Future studies are encouraged to investigate the individual inhibitors of cereal lipids and their inhibition mechanism.

**TABLE 4:** A summary of newly discovered lipids as α‐glucosidase and α‐amylase inhibitors

No.	Variety	Part used	Active compounds	Inhibited enzyme	Enzyme origin	Method	IC50	Reference
1	Wheat	Bran	1,2‐Dilinoleylglycerol‐3‐phosphate	α‐Glucosidase	Baker's yeast	pNPG	38.9 μM	Li et al. (2009)
		Bran	1‐Palmitoyl‐2‐linoleoyl glycerol‐3‐phosphate	α‐Glucosidase	Baker's yeast	pNPG	47.9 μM	
2	Wheat	Bran and germ	1,2‐Dilinoleylglycerol‐3‐phosphate	α‐Glucosidase	Baker's yeast	pNPG	27.1 μg/ml	Liu et al. (2011)
		Bran and germ	1‐Palmitoyl‐2‐linolery glycerol‐3‐phosphate	α‐Glucosidase	Baker's yeast	pNPG	32.2 μg/ml	
3	Wheat	Bran	Alkylresorcinols	α‐Glucosidase	Rats’ intestine	pNPG	37.58 μg/ml	Tu et al. (2013)

### 4.5 Crude extracts

Distinguishable differences among cereal species were proved based on the results of inhibitory activities of the cereal crude extracts against these two starch digestive enzymes (Table 5). Kim et al. (2011) studied the inhibition of α‐glucosidase and α‐amylase by 70% ethanol extracts of different varieties of sorghum, foxtail millet, and proso millet. The results showed that in 6 sorghum varieties, compared with the existing α‐glucosidase inhibitor anti‐diabetic acarbose (IC_50_ = 2.1 μg/ml), Mongdang‐susu (SS‐1), Me‐susu (SS‐2), Susongsaengi‐Susu (SS‐3), and Sikyung‐Susu (SS‐4) extracts had significantly higher inhibitory activity against α‐glucosidase (IC_50_ = 1.1–1.4 μg/ml). Moreover, these extracts have a strong inhibitory effect on α‐amylase in pancreas and saliva, while foxtail and proso millets extracts have no significant inhibitory effect on α‐amylase or α‐glucosidase activity. Ramakrishna et al. (2017) screened the antihyperglycemic function of 13 barley varieties and evaluated the α‐amylase and α‐glucosidase inhibitory activities of barley extracts (hot water, cold water, and 12% ethanol). The results showed that the cold water and ethanol extracts of most barley varieties had significant inhibitory effects on α‐amylase, but the differences among most varieties were not significant. Additionally, it was observed that for all extraction methods, the black barley variety had the highest α‐glucosidase inhibitory activity (34%) and exhibited a dose‐dependent pattern among all barley varieties. Ranilla et al. (2009) used in vitro enzyme assays to determine the α‐glucosidase and α‐amylase inhibition associated with early type 2 diabetes in 10 kinds of Peruvian Andean hot‐processed cereals (five cereals, three pseudocereals, and two legumes). The results showed that the purple corn (Zea mays L.) water extract had the highest α‐glucosidase inhibitory activity (51%, 5 mg sample weight). No α‐amylase inhibitory activity was observed in all of the evaluated Andean grains.

**TABLE 5:** A summary of newly discovered crude extracts as α‐glucosidase and α‐amylase inhibitors

No.	Variety	Part used	Active compounds	Inhibited enzyme	Enzyme origin	Method	IC50	Reference
1	Purple pigmented	Kernel	Water extracts	α‐Glucosidase	Baker's yeast	pNPG		Ranilla et al. (2008)
	Yellow, purple‐red mottled	Kernel	Water extracts	α‐Glucosidase	Baker's yeast	pNPG		
	Yellow, red blotched	Kernel	Water extracts	α‐Glucosidase	Baker's yeast	pNPG		
	Half yellow‐half red	Kernel	Water extracts	α‐Glucosidase	Baker's yeast	pNPG		
	Purple mottled	Kernel	Water extracts	α‐Glucosidase	Baker's yeast	pNPG		
2	Sorghum (Mongdang‐susu(SS‐1))	Whole grains	Ethanol extracts	α‐Amylase	Porcine	DNSA	4.5 ± 0.0 μg/ml	Kim et al. (2011)
				α‐Amylase	Saliva	DNSA	6.1 ± 0.4 μg/ml	
				α‐Glucosidase	Bacillus stearothermophilus	pNPG	1.1 μg/ml	
	Sorghum (Me‐susu(SS‐2))	Whole grains	Ethanol extracts	α‐Amylase	Porcine	DNSA	2.9 ± 0.5 μg/ml	
				α‐Amylase	Saliva	DNSA	4.5 ± 0.2 μg/ml	
				α‐Glucosidase	Bacillus stearothermophilus	pNPG	1.2 μg/ml	
	Sorghum (Susongsaengi‐susu (SS‐3))	Whole grains	Ethanol extracts	α‐Amylase	Porcine	DNSA	11.8 ± 0.1 μg/ml	
				α‐Amylase	Saliva	DNSA	10.3 ± 0.0 μg/ml	
				α‐Glucosidase	Bacillus stearothermophilus	pNPG	1.3 μg/ml	
	Sorghum (Sikyung‐susu (SS‐4))	Whole grains	Ethanol extracts	α‐Amylase	Porcine	DNSA	9.0 ± 0.4 μg/ml	
				α‐Amylase	Saliva	DNSA	10.2 ± 0.4 μg/ml	
				α‐Glucosidase	Bacillus stearothermophilus	pNPG	1.4 μg/ml	
	Sorghum (Jangsu‐susu (SS‐5))	Whole grains	Ethanol extracts	α‐Amylase	Porcine	DNSA	194.1 ± 3.1 μg/ml	
				α‐Amylase	Saliva	DNSA	333.3 ± 5.0 μg/ml	
				α‐Glucosidase	Bacillus stearothermophilus	pNPG	20.4 μg/ml	
	Sorghum (Heuin‐susu (SS‐ 6))	Whole grains	Ethanol extracts	α‐Amylase	Porcine	DNSA	>666 μg/ml	
				α‐Amylase	Saliva	DNSA	>666 μg/ml	
				α‐Glucosidase	Bacillus stearothermophilus	pNPG	102.7 μg/ml	
3	13 kind of barely	Seed	Hot water, cold water, and ethanol extracts	α‐Amylase		DNSA		Ramakrishna et al. (2017)
				α‐Glucosidase		pNPG		

## 5 CONCLUSIONS

**FIGURE 3:**
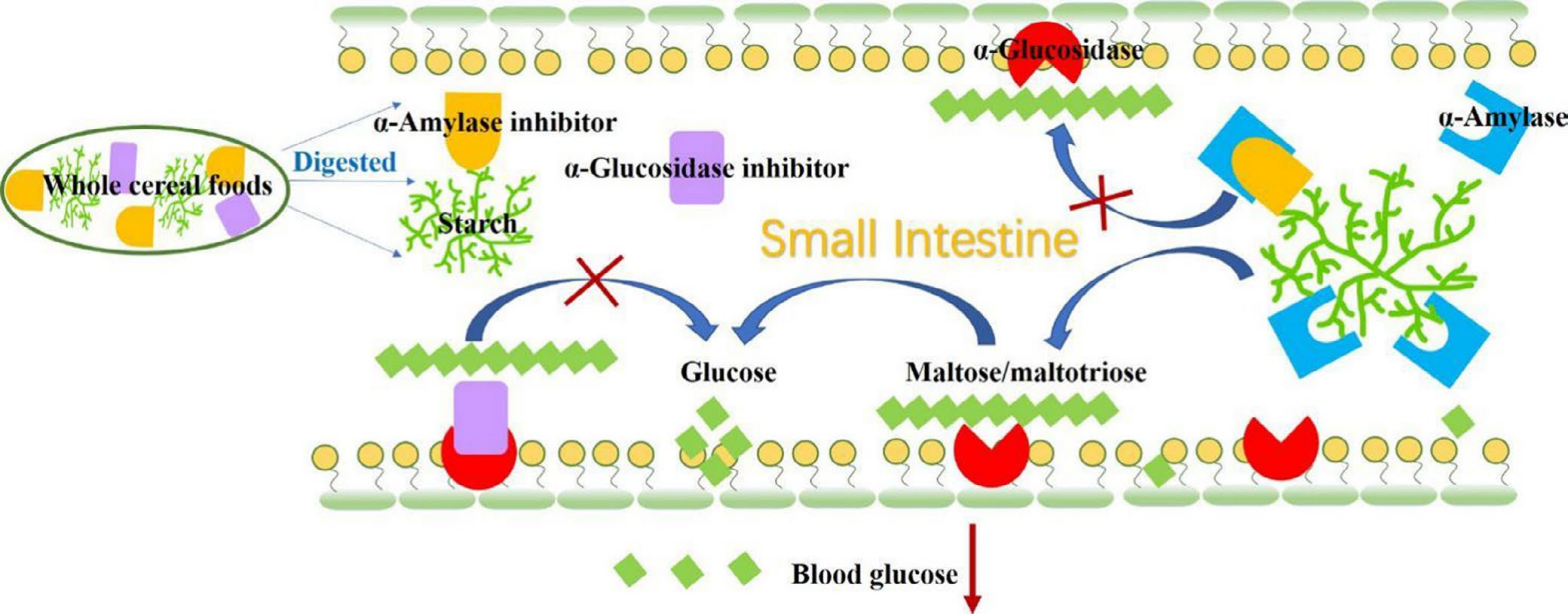
Potential mechanisms under the whole cereals on the prevention of hyperglycemia. The α‐amylase and α‐glucosidase inhibitors in whole cereal foods are released during digestion in the gastrointestinal tract. α‐Amylase inhibitors may limit the hydrolysis of starch by block the active centers of the enzymes. α‐Glucosidase inhibitors reduce shorter oligomer by occupying enzyme and sugar‐binding sites, thereby delaying intestinal absorption of glucose

The increased interest in whole cereal foods has coincided with an increase in the prevalence of chronic diseases such as T2DM. α‐Amylase and α‐glucosidase inhibitors are significant for the control of postprandial blood glucose in diabetic patients. Cereal‐derived phenolic compounds, peptides, nonstarch polysaccharides, and lipids inhibit α‐amylase and α‐glucosidase activity. These inhibitors may be associated with the prevention of hypoglycemia by whole cereal food intake (Figure 3). To increase the utilization of whole cereals and their bioactive ingredients in diabetes management foods, it is mandatory to understand the inhibitory mechanisms and further investigate the structure–activity relationships between the compounds and enzymes. For example, the number and location of hydroxyl groups of phenolic acids, the molecular weight of polysaccharides, acetylation, and methylation all affect the enzyme inhibition activities. Of note, processing technologies will be able to affect the distribution, compounds, chemical structures, amount, and thus health benefits of the end whole cereal food subject to cereal types. The cereal‐derived α‐amylase and/or α‐glucosidase inhibitor could be targeted for developing valuable whole cereal foods in T2DM dietary managements. Noteworthy, most data about the impact of whole cereal flour or whole cereal food on enzyme activities are from in vitro studies, and further in vivo investigations are needed.

## CONFLICT OF INTEREST

The authors have declared no conflict of interest.

## Data Availability

Data sharing is not applicable to this article as no new data were created or analyzed in this study.
